# Difficult Capacity Cases—The Experience of Liaison Psychiatrists. An Interview Study Across Three Jurisdictions

**DOI:** 10.3389/fpsyt.2022.946234

**Published:** 2022-07-11

**Authors:** Nuala B. Kane, Alex Ruck Keene, Gareth S. Owen, Scott Y. H. Kim

**Affiliations:** ^1^Department of Psychological Medicine, Mental Health, Ethics and Law Research Group, Institute of Psychiatry, Psychology and Neuroscience, King's College London, London, United Kingdom; ^2^Department of Bioethics, Clinical Center, National Institutes of Health, Bethesda, MD, United States

**Keywords:** decision-making capacity, liaison psychiatry, mental capacity, informed consent, capacity evaluation, capacity assessment, clinical ethics

## Abstract

**Background:**

Assessment of capacity for treatment and discharge decisions is common in the general hospital. Liaison psychiatrists are often asked to support the treating medical or surgical team in difficult capacity assessments. However, empirical research on identification and resolution of difficult capacity cases is limited. Some studies have identified certain patient, decisional, and interpersonal factors which cause difficulty, but no study has explored how these issues are resolved in practice. Our study therefore aimed to describe how experienced liaison psychiatrists identify and resolve difficult capacity cases in a general hospital setting.

**Methods:**

We carried out semi-structured interviews with 26 liaison psychiatrists from England, Scotland, and New Zealand, on their most difficult capacity cases. Thematic analysis was used to examine types of difficulty and how these were resolved in practice. Summaries were prepared and example quotes extracted to illustrate phenomena described.

**Results:**

We identified four types of difficulty in capacity assessment, spanning both clinical and ethical domains: 1) Difficulty determining whether the decision is the patient's own or driven by illness, 2) Difficulty in applying ethical principles, 3) Difficulty in avoiding personal bias, and 4) Procedural difficulties. The liaison psychiatrists presented as self-reflective and aware of challenges and pitfalls in hard cases. We summarized their creative strategies to resolve difficulty in assessment.

**Conclusion:**

Practitioners approaching difficult capacity cases require both clinical skills, e.g., to uncover subtle illness impairing decision-making and to consider interpersonal dynamics, and ethical skills, e.g., to negotiate the role of values and risks in capacity assessment. Education and training programmes should incorporate both aspects and could include the resolution strategies identified in our study. Practitioners, supported by health and social care systems, should work to develop self-aware and reflective capacity assessment practice.

## Introduction

Assessment of decision-making capacity (henceforth capacity) is common in the general hospital (1), where capacity for treatment and discharge decisions can be impaired by a wide spectrum of conditions including organic cognitive disorders and mental disorders (1–4). While capacity assessments are primarily carried out by the treating medical or surgical team (5), (consultation) liaison psychiatrists are often asked to provide a second opinion or to support the treating team to assess the patient's capacity, particularly for complex assessments, if mental disorder is suspected to impair decision-making, or where the therapeutic relationship is at stake (1, 6, 7). Yet some assessments can be difficult to judge for even ‘expert' capacity assessors, who have expressed perceptions that training in this area is sub-optimal (8).

Empirical research on identification and resolution of difficult capacity cases is limited. A Swiss interview study (9, 10) identified challenges in capacity assessment including patient factors (mild or fluctuating cognitive impairment, complex medical or psychiatric needs), decisional factors (high stakes treatment refusal, new living arrangements), and differing opinions between professionals or professionals and relatives. An English survey further identified decisions about life-sustaining treatment, and capacity cases with inaccessible facts, urgency, or undue influence, as particularly challenging (11). Wider literature has highlighted other difficulties, including presence of subtle mental disorder such as plausible delusions or mood-related optimism/ pessimism (12–15), decision-making impaired by impulsivity (16–18) or valuational factors (19, 20), underlying ethical (21) or interpersonal (7) issues, or when patients are angry or uncooperative (12, 14, 22). To date, no study has examined how such difficulties are resolved in practice by experienced practitioners.

Our cross-jurisdictional study undertook in-depth interviews with 26 experienced liaison psychiatrists for whom capacity assessments constitute a substantial part of their day-to-day clinical work in an inpatient medical setting. Our study is unique in its focus on experienced practitioners' self-identified hard to judge capacity cases and how they resolved these cases. Through probing how experts make capacity judgments in hard cases, we aimed to describe the attitudes and practices underlying such judgments, including norms around decision-making capacity and its application.

## Methods

### Setting and Sample

Semi-structured in-depth interviews were carried out with 26 liaison psychiatrists across three jurisdictions. Participants were recruited by purposive sampling, through liaison psychiatry email lists and presentations by the researchers at conferences. We chose purposive sampling with the aim of achieving depth of understanding of hard capacity assessments in the general hospital setting (23). We selected liaison psychiatrists, with a) substantial clinical experience of carrying out or supervising capacity assessments (consultant level) and, b) familiarity with capacity law (self-reported). We selected liaison psychiatrists because the legal framework in the general hospital involves mental capacity law more than mental health law. We recruited from England, Scotland, and New Zealand because these are English-speaking jurisdictions, with broadly similar capacity regimes, where legal debates on capacity have been prominent over the past decade. Sampling was completed when the team judged that data saturation was reached, defined as no new themes emerging. Ethical approval for the study was obtained through King's College London Research Ethics Committee [see Supplementary Material 1 for the study's consent form and Supplementary Material 2 for the participant information sheet].

### Data Collection

The interviews with liaison psychiatrists were carried out in person, at hospitals where the interviewees practiced, or *via* video call. The interview protocol was developed collaboratively by the co-authors (who have backgrounds in psychiatry, law, and bioethics) and adapted for each jurisdiction. Prior to interview, interviewees were given a short brief asking them to recall one or two capacity assessments where they found it difficult to judge whether the person had or lacked capacity for the specific decision. The interview protocol (see Supplementary Material 3) covered probes for each hard case: the decision (context, options, risks), mode of assessment, suspected impairment, the patient's wishes and values, and how the case was resolved, as well as general questions on difficulty, unwise decisions, and objectivity. Interviews were conducted between April and August 2018. NK led all interviews, with a co-author present for 5 of 26 interviews. She was, at the time of the interviews, a post-membership psychiatrist with clinical experience in liaison psychiatry and academic experience of analyzing court judgments on capacity. Duration of interview was approximately 60 min. Interviews were audio recorded and transcribed, with transcripts stored securely in anonymized form, alongside field notes.

### Analysis

Thematic analysis was chosen due to its flexibility and rigor, allowing description (and interpretation) of qualitative data in rich detail (24). Transcripts were uploaded to NVivo software. NK read all transcripts, generated and applied codes, and organized codes into themes and subthemes in an iterative, inductive process. All co-authors read a proportion of the transcripts (SK read 13 of 26 transcripts while ARK and GO each read 8 of 26) and met regularly for interval discussions during the analysis; after each round of reading and discussion, the thematic map was reviewed and updated. The analysis resulted in four categories of factors causing difficulty in capacity assessments, with subcategories. Summaries were prepared for each subcategory and example quotes extracted to illustrate phenomena described. Detail of the psychiatrists' approaches to resolution of each difficulty, where available, was reported in the relevant subcategory. Extracted clinical material was checked for anonymity and interview participants were given the opportunity to review clinical material. The ‘standards for reporting qualitative research' (SRQR) reporting guidelines (25) were used in reporting this study (see Supplementary Material 4).

## Results

### Participant Characteristics

Table 1 outlines characteristics of the 26 consultant liaison psychiatrists interviewed, including jurisdiction of practice (13 in England, 10 in Scotland, 3 in New Zealand), age, gender, ethnicity, clinical subspecialty or special interest(s), and extra-curricular activities related to capacity. When asked how often they dealt with referrals for capacity assessment (personally or *via* supervision of junior staff), 14 reported weekly or more frequently (with five reporting daily frequency), two reported monthly, four reported that capacity assessments made up 10–30% of referrals to the liaison service, and others identified such referrals as “very common”, arising “a lot”, or “quite central” to their clinical practice. A total of 73 relevant hard capacity cases were shared, a mean of 2.8 cases per psychiatrist.

**Table 1:** Characteristics of Liaison psychiatrist interviewees.

Jurisdiction	England	13
	Scotland	10
	New Zealand	3
Gender	Male	16
	Female	10
Ethnicity	White	21
	Asian or mixed Asian	4
	Black	1
Age	36–64 years (mean 47)	
Liaison psychiatry subspecialty or special interest(s)^a^	Older adult liaison psychiatry (psychogeriatrics)	8
	Transplant/ renal psychiatry	6
	Neuropsychiatry/ Huntington's disease	4
	Medically unexplained symptoms	4
	Other^b^	6
Extra-curricular activities related to capacity^c^	Independent or court assessments	13
	Academic or policy activities	7

a *23 of 26 psychiatrists worked in general adult liaison psychiatry (with or without subspecialty interests); 2 worked exclusively in older adult liaison psychiatry; 1 worked exclusively in liaison neuropsychiatry*.

b *The other category included special interests in psychiatry of eating disorders, HIV, pain, psycho-oncology, and obstetrics*.

c *This was not explicitly probed at interview but several participants spontaneously referred to capacity-related activity outside of their usual clinical practice. Independent assessments included Deprivation of Liberty Safeguards (DoLS) assessments and Human Tissue Authority assessments of living organ donors*.

### Thematic Analysis

#### Theme A. Difficulty Determining Whether the Decision Is the Patient's Own or Driven by Illness

##### A1. Is the Unusual Decision Due to a Hidden Delusional Belief or Cultural Factors?

- *P9: So I think that was one of those things where the whole capacity assessment could be… could hinge on whether something was a psychopathology, or whether it was a widely held belief that was shared by lots of people, and quite understandable in the context of someone's history [and] biography*.

Several hard cases hinged on the nature of the beliefs driving the patient's treatment refusal, where there was difficulty distinguishing whether these were due to a delusion or whether they were religious, cultural, or political beliefs. This was because (a) the relevant delusion was hidden or subtle, or (b) it was hard to tell if beliefs (unusual or unfamiliar to the psychiatrist) were congruent with the person's own personal or cultural belief system. In resolving these cases, attention to psychopathology as well as to the patient's personal history and culture (often through involving others from the same culture) was useful. In one case, an elderly patient was refusing painkillers on religious grounds, initially (and mistakenly, as it turns out) raising the question of whether there might be a psychotic illness impairing his decision-making. The psychiatrist explored his personal history, learning that the patient had been staunchly religious throughout his life, and invited a priest to meet him and give a view. The priest's view was that the patient's decision was in keeping with his faith, and the patient was deemed to have capacity. In contrasting cases, it was possible to delineate cultural factors and reveal subtle delusions driving decision-making. One such example involved a patient who embarked on a hunger strike on ostensibly political grounds, citing persecution in his country of origin. Through careful examination of his history and mental state over multiple assessments, the psychiatrist uncovered a persecutory delusional disorder, confirming a (temporary) lack of capacity to refuse feeding. He was fed with a nasogastric tube and, as his delusions resolved with antipsychotic medication, his capacity improved and he ceased his hunger strike.

##### A2. Is the Decision Impaired by Depression or Is It ‘Perfectly Understandable'?

- *P1: But actually the disagreement was partly held around that “Is he depressed, or is he just making a decision that's perfectly understandable, that all of us would make if we had such a terminal, serious illness?”*

Several psychiatrists discussed treatment-refusal cases which raised the question of whether the patient had a depressive illness which impaired their ability to value their life or their future. Difficulty arose when the patient's depressive illness was of unclear severity and their quality of life was impaired by other factors, for example, an older patient with chronic kidney disease who expressed the wish to stop renal dialysis. Resolving such cases was complex, and psychiatrists raised the following questions: whether the decision-making seemed “understandable” or “reasonable” (considering their judgments of the patient's quality of life), the duration of the patient's outlook and whether this might be responsive to intervention, how the psychiatrist might evaluate similar circumstances, and finally, presence of emotional symptoms of depression or pervasive suicidality. One psychiatrist linked the difficulty in judging capacity in these cases to a difficulty inherent in diagnosing depression, specifically in disentangling illness from an understandable response to difficult circumstances.

##### A3. Are Extreme and Transient Emotions Driving the Decision, Causing Incapacity?

- *P4: So this is where you start to say, is someone sat in front of you with tears streaming down their face, you know, sobbing away, “Why doesn't he/she love me anymore?” Is that an impairment of mind or brain? Is extreme anxiety, anger, distress an impairment of mind? I mean, you could easily argue that it could be, dependent on what you're actually needing to decide at the time*.

Psychiatrists reflected on whether and how extreme emotions could cause incapacity. Several psychiatrists discussed assessments in the emergency department for patients refusing treatment following overdose. Others raised treatment refusal following bad news, such as life-changing spinal injury or new diagnosis of metastatic cancer. The connection between extreme emotions and mental disorders was also discussed; phobias were raised, as was borderline personality disorder, a condition characterized by emotional dysregulation and impulsivity. However, psychiatrists cautioned against attributing mental disorder to all patients in distress.

Some psychiatrists argued that emotional patients could still make decisions right for them and raised concerns about a slippery slope to paternalism in overruling emotional decisions. However, in cases of decisions with irreversible consequences, some psychiatrists felt anxious about patients changing their mind when emotions had cooled. To resolve these cases, one psychiatrist spoke of carrying out an in-the-moment assessment of how overwhelmed the patient was by their emotions and whether they felt it likely that the patient's wishes would change. Other approaches included to delay the capacity assessment (where possible), involve someone with a different skill set, gather information on whether decision-making was inconsistent with the person's values, and in some cases to act pre-emptively, e.g., offering psychological interventions to pregnant patients with needle phobia and collaboratively preparing advance directives regarding life-saving treatment while giving birth.

##### A4. Is There a Real or Just Apparent Lack of Insight?

- *P17: If this lady says, “I don't have HIV”, is she cognitively simply so impaired that she cannot compute this, or because she does not want to have this disease, she says “I haven't got HIV”?*

Several cases touched on whether a patient lacked insight into their condition or care needs to the extent that they lacked capacity. Psychiatrists grappled with whether a lack of insight was due to some pathology or only an apparent deficit reflecting a recognizable human reaction (or both). In the quoted case of a woman with HIV encephalopathy refusing treatment, the psychiatrist cited her personal history of high-status relationships and wondered about HIV as a threat to her self-conception and identity. In another case, a patient with Huntington's Disease had a flippant attitude to her care, which might have represented an organically based anosognosia or a defense mechanism borne of seeing relatives suffer with the same condition. Psychiatrists differed on whether a psychological process of denial (in the absence of an organic correlate) could impair capacity, with one arguing that strong psychological denial could do so. They also expressed concern about whether patients might be (understandably) minimizing their difficulties in the presence of strong values, for example, to live in their own home, or might be reticent about speaking openly to their assessor about their difficulties, rather than truly lacking insight due to cognitive impairment or mental disorder. In resolving these cases, clarifying the severity of cognitive impairment or mental disorder was helpful.

##### A5. Is Superficial ‘High Functioning' Masking Impaired Decision-Making?

- *P17: when you just superficially engaged with him then you thought, “Well he knows what he's talking about; why are we assuming here that he lacks capacity?” It was important to look at the difference between what he was actually saying and what he was doing*.

Several cases involved patients with good verbal functioning, who could “*talk a good game*” [P24] but lacked the ability to follow through on their statements in reality. Typical cases involved care refusal by patients with alcohol-related brain damage or subcortical dementias, with associated executive dysfunction (impaired planning, initiation of and persistence with tasks) and impulsivity. Psychiatrists recognized that non-specialist or brief assessments struggle to identify such difficulties. In resolving these cases, several psychiatrists recommended gathering evidence on the person's real-world functioning and putting this information to the person at interview. Often several assessments were necessary. The psychiatrists also found it helpful to compare current and premorbid decision-making, e.g., a previously meticulous patient with Huntington's Disease began to make reckless decisions on fire safety at home, suggesting a new lack of capacity around residence decisions; in cases where patients had always been impulsive, matters were more complicated. Several psychiatrists cited findings from neuro-imaging or neuropsychological testing as helpful in providing causal explanation for the impairments underlying the incapacity.

##### A6. Is a Personality Disorder Driving the Decision, and Has This Caused Incapacity?

- *P23: His history was absolutely pathognomonic of a paranoid personality disorder […] The way I felt he was weighing [the treatment decision] up was that his overdeveloped sense of personal rights and his, essentially, need for conflict, and to have a battle, and to win the battle was such that […] it had actually become more important to him to win that battle than to have a good outcome. And he was […] giving huge weight to that in his decision-making process. And my feeling was that... that was him; that's what he did*.

Several psychiatrists raised cases of seemingly erratic or unusual decision-making in patients with unusual personalities raising alarm. However, they gave considerable leeway to ‘unwise' or ‘imprudent' decision-making where they felt this was consistent with enduring patterns of (even maladaptive) behavior. In the above quoted case, a patient with chronic pain and loss of function from an old leg injury requested an amputation, while his orthopedic surgeon favored conservative treatment but did not rule out amputation. On assessment, the psychiatrist felt the patient's weighing, while unusual, was in keeping with his personality, values, and world view, and that he had capacity to opt for surgery. He had the amputation and did very well, as “*he was highly motivated to prove that he was right to demand the amputation so worked really well with the [rehabilitation] service”*. In similar cases, resolution was achieved, not only by history-taking, but through consulting families about whether a decision was in keeping with a patient's character, with one psychiatrist stating: “*often [the family] say, well they're very upset but they're not really surprised [by unusual decision-making]* [P14]. However, some argued that some personalities could be so extremely unusual as to render someone, in specific situations of unusual stress, unable to weigh up information for certain decisions. Others cited an increased vulnerability to other mental disorders and a need for assessment to be time-specific.

#### Theme B. Difficulty in Application of Ethical Principles

##### B1. Protection of Welfare

- *P14: … you tend to get called for those particular ones where the stakes are far higher, where it's often a life and death decision, and then you really don't want to be casual about how you assess someone's ability to use and weigh information, because on the one hand you don't want to be too coercive, and on the other hand you don't want just to let someone's life slip away by making the wrong judgment*.

Psychiatrists identified that difficult capacity assessments tend to involve high risk or ‘high stakes' decisions, for example, refusal of life-saving treatment or discharge home alone with high care needs or falls risk. Several gave a substantive view of risk-sensitive capacity assessment, i.e., that higher risk decisions led to an increase in the capacity threshold or cut-off, such that the higher the risk the greater the capacity required to make the decision. One psychiatrist characterized this as a ‘sliding scale' model, and others spoke about ‘setting the bar' and ‘drawing' or ‘shifting' the threshold.

One such case involved a woman with anorexia nervosa and swallowing dysfunction who was first deemed (by another assessor) to lack capacity to decide what to eat. Subsequently, after the speech and language therapist clarified that choking risk from certain foods was less than thought, she was deemed by the psychiatrist to have capacity. While other supportive interventions were taken in the interim (introducing greater flexibility around mealtimes), the shift in risk was also felt to be significant to the new capacity judgment.

Some psychiatrists did not question a ‘sliding scale' approach and indeed more than one referenced supportive literature. Other psychiatrists felt more uncomfortable, including reflecting on interaction with the presumption of capacity. They worried that, e.g., for a patient with cognitive impairment making a risky decision, the onus shifted to the patient having to prove their capacity rather than the psychiatrist having to prove incapacity.

Others described an epistemic risk-sensitivity, whereby a higher risk demands greater evidence of intact capacity. Despite these differences, there was broad consensus that increasing risk places an onus on the psychiatrist to carry out a more intensive assessment and to ensure all avenues were explored to support and engage the patient.

##### B2. Autonomy

When making a capacity determination involves room for judgment, as is common in difficult cases, the psychiatrists recognized the influence of societal emphasis on autonomy. For example, in resolving the case of a patient with borderline personality disorder who decided to cease chemotherapy (and was found capacitous), the psychiatrist raised prevailing societal views:

- *P14: I suppose the general public as well have a view that they see autonomy of choice as above every other, if you like, ethical consideration. And I think as a profession we've stepped back from a paternalistic role, in line with, I suppose just what the general public expect and demand now […] we're very much their servants […] their autonomy is certainly sacrosanct and their capacity is presumed*.

However, how to approach questions of autonomy was not always clear-cut, for example, in end-of-life cases. In the case of a physically unwell patient who was refusing to eat, the psychiatrist spoke about competing paradigms around dying for people with physical illness, contrasting a ‘zero suicide' framework, a palliative care framework (where death is to be accepted and planned for), and an assisted dying framework (where one can choose the time and means of one's own death):

- *P7: And I think that where it becomes the most tricky is where we've got somebody who is dying, perhaps even imminently dying, and that their disease will kill them, yet they're talking about it being too much and wanting to kill themselves, or thinking about killing themselves, or ending their lives more quickly than they would. And then we're sort of… we have to then assimilate all of those different paradigms, and how acceptable they are, in one person, in one assessment*.

Here, the ‘acceptable' degree of autonomy (around one's own death) was dictated by competing frameworks, and the psychiatrist felt pushed to act as a kind of arbiter between them. This difficulty was not easily resolved—the psychiatrist postponed capacity assessment and focused on supporting the patient.

##### B3. Justice

Difficulty also arose when ethical principles other than autonomy or welfare intruded on the assessment. Two psychiatrists spoke about assessing capacity in patients who had organ transplants – both were mindful of the value placed on this ‘precious resource' and the added ethical implications in terms of justice if a patient who had accepted a transplant was now refusing food or medical treatment. A drive to protect the transplant organ, which could have gone to someone else, was experienced as adding to complexity, although not permitted to determine capacity.

##### B4. Non-discrimination

Psychiatrists raised concern about the potential for patients with mental disorders to be allowed less scope to make eccentric decisions than those without mental disorders:

- *P15: I don't have to tell anybody how I made the decision to spend a thousand pounds on a handbag if that's what I decide to do; no one questions my capacity […] But if you've got autism, the minute you decide to make that decision someone questions it*.

However, in one case, these concerns led a psychiatrist to reflect on the boundaries of mental disorder, as an alternative approach to resolution. In this example, the parents of a young man with Asperger's Syndrome raised concern about his ability to manage his finances. The psychiatrist argued, however, that the patient's way of managing money was just different, not incapacitous, and commented: “*I don't see [Asperger's] as a mental illness, I see it as a different way of being”* [P8]'.

#### Theme C. Difficulty in Avoiding Personal Bias

##### C1. Countertransference/ Clinician Attitudes Toward Patients

Several hard cases, in the view of the psychiatrists, involved the referring team's bias of desiring a certain clinical outcome (e.g., to keep or discharge the patient) and hence a certain capacity judgment. Sometimes the team wanted to act in a paternalistic way, for example, in the case of a motherless young patient with a chronic illness who the team had cared for over many years and, in effect, assumed a kind of parental role for him. The question was whether he could decide around end-of-life arrangements. The psychiatrist suspected that an incapacity finding was preferred “*because then [the team] could make the decisions for him in our sort of looking after him kind of way”* [P3]. The psychiatrist identified that the challenge was not really in determining the patient's capacity but rather in supporting the team to accept his decisions and his death.

In contrast, sometimes a patient was angry, antisocial, or difficult to manage on the ward, generating a strong negative emotional response in the treating team. One psychiatrist reflected on patients, whose decision-making was likely impaired through drug intoxication and withdrawals, who were permitted to self-discharge without referral to liaison psychiatry:

- *P15: Oh I think no one wants to treat an angry antisocial person. I mean… sorry, I say nobody… people do their very best to treat them but when they become difficult, if there's an opt-out of some sort, sometimes it's very easy to let that group of patients go*.

In such cases, the psychiatrist was able to resolve the difficulty by identifying and reflecting the team's bias and reorienting them to the patient (whose capacity then tended to be easy to judge).

##### C2. Professional or Personal Commitments

Psychiatrists were also willing to admit their own biases in assessing capacity, including emotional responses to suffering and risk, professional fears and values, and personal political views, as well as suggesting ways of minimizing bias to resolve hard cases.

- *P15:...But I think because we are human and we have emotional connections with the scenarios that we're in, it's really hard to be fully objective about capacity. And again, I think because people are easily alarmed by risk and life or death consequences, that arouses a certain amount of concern that then clouds your judgement*.

Some reflected on the emotional toll of seeing patients whose decisions caused their welfare to deteriorate or, conversely, whose autonomy was forcibly restricted through coercive treatment. Others spoke frankly about their fear of litigation or of being criticized in the event of the patient coming to harm. Others reflected on their own personal political views, with one self-identifying as “fairly libertarian” and another stating: “*I know that I have a much lower threshold for saying somebody's capable than many of my colleagues, and a much higher threshold for saying they're incapable […] I think it's because I personally value autonomy as an ethical principle possibly more than my colleagues.”* [P13] Professional differences were also raised, with the medical profession felt to be more inclined than social workers to find patients lacking capacity in borderline cases. Various explanations were suggested, including that doctors more vividly understand the potentially grisly consequences of untreated illness or have an instinct to care for people or to “do something”.

Several psychiatrists counseled how easy (and so, dangerous) it is to “*set the anchor pretty close to your own perspective”*[P14] or assume one's own value framework is a standard against which a patient's decision-making should be judged. They advocated self-awareness and cited cases where resolution required second opinion assessments, multidisciplinary discussions, or informal discussion with colleagues. Divisive issues were identified as particularly vulnerable to bias, including end-of-life care and late termination of pregnancy. One psychiatrist spoke of purposefully stepping back from assessments where their own views were too strong:

- *P6: my own religious, personal, moral, ethical beliefs—call them what you will—will undoubtedly color a capacity assessment regarding late termination [of pregnancy…] So I wouldn't do it. Although there might be a temptation in me to do it because then I think I'm saving a baby's life. But I've got to try and be professional and stand back*.

Here, the psychiatrist felt that resolution was best achieved by asking a colleague to carry out the assessment instead.

#### Theme D. Procedural Difficulty

##### D1. Lack of Engagement

Often difficulty arose because a patient refused to engage in the capacity assessment or engaged in a limited manner only. For example, a patient refusing treatment was referred due to concerns that depression was impairing his decision-making. On assessment, the psychiatrist found:

- *P14: He understood perfectly well what the purpose of each of my questions was, in trying to delineate his capacity or whether he was depressed, because it is, in some respects, fairly transparent to most people what it is you're asking them to do. And he played it with a dead hand, “No, no, no… yes, no, no…”, and so there was nowhere to go with it; he wouldn't really engage in the conversation; there was no meaningful discussion*.

The inability to establish “meaningful” discussion meant the assessment had a superficial quality. The psychiatrist judged that the patient had capacity but with a lingering sense of discomfort: “*I think he just saw me as another, so to speak, instrument of the State, [there] to try and thwart a decision that he wanted to take”*.

Another patient, refusing urgent surgical treatment, was willing to engage on all subjects except for her reasons for refusing surgery, leaving her family and professionals feeling uneasy, even while she was judged to have capacity. To resolve this case, the psychiatrist reviewed the patient multiple times to ensure confidence that no mental disorder was impairing decision-making. They also shared a psychological formulation for why the patient was withholding information in this way, attributing this to relationship dynamics and anger about other issues in her life.

In a contrasting case, a psychiatrist judged a patient to be unable (due to a psychotic disorder) rather than unwilling to engage in the assessment of her capacity for care decisions. Through building rapport over time, the psychiatrist established that a “dismissive” exterior masked a persistent and pervasive poverty and inflexibility of thought which impaired decision-making. In similar cases, observations by the multidisciplinary team were also cited as helpful to resolution.

##### D2. Lack of Information to Verify Facts

Several psychiatrists made the point that relevant information on the facts of the person's situation could be difficult to clarify, particularly for decisions around discharge planning (care and residence). The lack of verified information can make assessments difficult:

- *P21: [Welfare concerns can be] less specific, so how do I know that he's acknowledging just how dirty his flat is? And even food, you know, you'd get into these vague conversations about, “Oh but I did drink that milk and I went out to get milk afterwards”. “But the nurse said the milk was still this date” […] In our position, of just being hospital based and kind of it's all a bit retrospective, and it's based on the home-help who works for an agency that I'm getting third-hand [information], vs. the patient who is… it's difficult. I mean, it's do-able but I suppose with our resources it's much more difficult to get a solid… solidly test their capacity about that*.

To resolve this case, the psychiatrist contacted community services directly but acknowledged that time and resources were an issue. The issue of missing information also came up in financial capacity assessments and in assessing “out of area” patients without local records.

##### D3. Interpersonal Conflicts Between Patient and Team

Sometimes psychiatrists are consulted for capacity to find the patient entrenched in conflict with the treating team. Often these patients are capacitous and the issue is predominantly interpersonal. The psychiatrists spoke about patients, aware that the medical team disagreed with their wishes, digging their heels in and holding their position more firmly:

- *P3: I think for the patient sometimes they become quite… defensive […] They realize that […] the medics as a group disagree with their wishes, and so they become quite defensive about why they've made that decision [and] immediately, you know, you have to break down a lot of those barriers*.

Some psychiatrists spoke about the role of “face-saving” in such cases, where escalating interactions with the clinical team have left the patient with no way out of the conflict without losing face.

Sometimes the psychiatrist understood treatment refusal as a kind of protest or a way for the patient to bargain in relation to treatments offered or to regain control over their treatment:

- *P14: So sometimes it's… a “protest” against just being caught in a system over which they have no control; the only time anyone sits up and takes notice is when they refuse to engage in it anymore. And that's the time when they start to get some sort of attention from a busy and over-pressed medical service. And so sometimes, you know, them saying that they don't want to take treatment anymore, and they want to end it all, is a very different communication. And sometimes when you get to the bottom of that, it's far more about them feeling entirely out of control. And then the resolution has been more about bringing them into the decision-making process and giving them control*.

In resolving such cases, the psychiatrists were mindful of their role as a new person who might be able to negotiate or mediate between the parties “at loggerheads”, to explore the patient's reasons for refusal, ensure they had been provided with adequate information, and support their involvement in their treatment and the decision-making process. Sometimes, introducing increased flexibility, for example through exploring alternative care options more in line with the patient's wishes, was helpful.

##### D4. When “Please Assess Capacity” Belies Therapeutic Concerns

Several psychiatrists raised hard cases where the capacity consult belied pressing therapeutic issues, such as the need for crisis intervention or consideration of whether the patient's wishes should be overruled (if incapacitous).

In some cases, a request for capacity assessment sat alongside a need for crisis intervention, with psychiatrists giving examples of patients with borderline personality disorder attending A&E following an overdose or self-harm but refusing emergency care on arrival. Some psychiatrists considered these patients to be ambivalent about treatment, and one formulated this in psychodynamic terms:

- *P26: And, you know, when I saw her, it was clear that she didn't want to die; she wanted to be looked after […] because she alerted services, and she made no attempt to resist coming into the ambulance. … you know, it's her experience of care as both therapeutic as well as abusive. And she's constantly in conflict about accessing care as well as refusing it. […] Which is sort of replaying what has happened to her in early life*.

Resolving these cases involved a therapeutic approach—psychiatrists emphasized the importance of validating the patient's distress, listening to their concerns, and instilling hope. One psychiatrist discussed how this therapeutic framework sat alongside the capacity framework:

- *P4: I'm coming in to do this assessment of a human being who's in crisis, who's trying to take their own life, or is trying to hurt themselves very badly, and they've got themselves in a real pickle. Let's leave capacity aside. I'm going to try and understand why, what's kind of going on here, you know… how can I actually help you, one human being to another, coming from a profession that way pre-dates imposition of a capacity framework. Now if that person continues, at the end of my assessment to say ‘no', I'll reintroduce the capacity framework. If they don't, then I'm not going to say I've manipulated them into a correct capacity decision; I'm going to say…, a capacity framework is not required here because the person is now saying, “I'll have treatment”*.

In other cases, the key therapeutic consideration was whether an incapacity judgment should lead to the patient's wishes being overruled. Sometimes, on discussion with the treating team, it became clear that overruling the patient was unsafe or impractical (e.g., proceeding with forced renal dialysis in the long-term), or because it would contravene patient dignity or the principle of the least restrictive option. In such cases, the capacity question became somewhat ‘academic', and psychiatrists saw less merit in pursuing a thorough capacity assessment:

- *P20: it's little bit like medical school… we're taught that for any tests that you do, ask yourself what are you going to do if the test is positive? And what are you going to do if the test is negative? And if they're the same thing, you don't do the test*.

Sometimes the psychiatrist supported the treating team to provide a flexible alternative, e.g., rather than a prolonged hospitalization and forced surgery for a paranoid patient with appendicitis, the team offered antibiotics and close outpatient monitoring with a good clinical outcome.

In some cases, the psychiatrist judged it counter-therapeutic to overrule the patient's wishes, emphasizing the importance of a patient's sense of agency in their recovery from illness. This concern sometimes led a psychiatrist to postpone a capacity assessment, or err toward findings of capacity, due to concerns about the impact of restrictive practice on the patient.

## Discussion

### Main Findings

#### Finding 1—Both Clinical and Ethical Skills Are Necessary in Hard Capacity Cases

The first main finding from our study is that liaison psychiatrists talk about four domains when asked about cases they find difficult to judge, two of which draw on clinical perspectives and skills (Themes 1 and 4) and two of which deal primarily with engaging with and/or adhering to ethical principles or values (Themes 2 and 3).

##### Clinical Skills

Firstly, clinical knowledge and skills were crucial to resolution of difficulty in determining whether a decision was the patient's own or driven by illness, as outlined in Theme 1. This finding is significant in the context of concerns expressed by legal scholars about over-reliance on psychiatry in capacity assessment (26, 27). Our study suggests that determining functional inability may require specialist skills to probe psychological processes and uncover subtle illness, for example, delusional disorders, depression, or executive dysfunction, impairing decision-making. The latter example chimes with recent literature advocating for specialist training in detection of ‘invisible' deficits which impair capacity in brain injury patients (16). Judges have expressed concerns about psychiatrists allowing specialist knowledge of illness to overshadow capacity assessments, e.g., *Re FX* [2017] EWCOP 36. However, in our study, psychiatrists also paid careful attention to the patient as an individual, their beliefs and values, personal and decision-making history, drawing on expertise of family and others. This approach is very much in keeping with that of the much-publicized Court of Protection case, *King's College Hospital NHS Foundation Trust v C* [2015] EWCOP 80, which found C to have capacity to refuse renal dialysis, giving particular weight to the evidence of C's daughters who strongly advocated that her decision-making was driven by “her value system and personality”. In attending to both illness and the individual in this way, the psychiatrists are essentially deploying the clinical method: obtaining history, mental state examination, collateral sources of information, and using knowledge of illness trajectories, to resolve an authenticity question: ‘is illness preventing this patient from making their own decision?'.

Secondly, as outlined in Theme 4, clinical knowledge and skills were useful in negotiating procedural difficulties in assessment, often related to the therapeutic context. Our study suggests that clear boundaries between capacity and therapeutic questions are not always possible in medical settings. For example, in treatment refusal cases involving patients who had self-harmed, the assessment process demanded psychotherapeutic skills to defuse the crisis and to work with ambivalence, rather than pursue immediate capacity assessment. In cases where endorsing a patient's agency was likely to bring therapeutic gains (28, 29), psychiatrists were reluctant to act in a way that might threaten the patient's agency. Such decisions to postpone capacity assessment, deploying therapeutic skills to support the patient in the interim, can be seen as fitting with the ‘support principle' of the Mental Capacity Act. But these examples also highlight clinical complexity and add nuance to debates about outcome concerns threatening the neutrality of capacity assessment (30–32). Clinicians, familiar with therapeutic goals and relationships, may be best placed to consider the role of the capacity assessment in the overall therapeutic landscape and negotiate these complexities.

##### Ethical Skills

Ethical knowledge and skills are no less important, as primarily evidenced by Themes 2 and 3. Firstly, our study shows that psychiatrists grapple with the risk-sensitivity of capacity assessment in practice. This is significant in context of previous findings that clinicians endorse risk-sensitive assessments, although with considerable individual variability (33) and with a discrepancy between general and case-specific attitudes (34). We noted that the way psychiatrists discussed risk reflects divisions in theoretical literature between advocates of substantive risk-sensitivity [where higher risk raises the threshold for capacity or its criteria e.g., understanding (32, 35)] and epistemic risk-sensitivity [where higher risk raises the requirement for evidence of intact capacity (31, 36, 37)]. Psychiatrists specifically raised concern about the interaction between risk-sensitivity and a presumption of capacity, one of the principles in the Mental Capacity Act and discussed in the literature (38, 39).

Secondly, our study showed that psychiatrists grapple with the role of emotions in capacity assessment, engaging with the normative question: ‘how emotional is too emotional to decide?' Their approaches to resolution, including considering how overwhelming the emotional state was or whether it caused the patient to make a decision out of keeping with their usual preferences, reflect procedural approaches in the literature (19). In contrast, hard cases involving insight did not reflect conceptual difficulty with insight in capacity assessment (40, 41) but rather practical difficulties in establishing whether an apparent lack of insight was real or not.

Thirdly, psychiatrists in our study showed awareness of both societal and personal values. There was evidence of fidelity to societal expectations of practice, for example, in terms of striving to respect autonomy. Psychiatrists also made efforts to mitigate the influence of their personal values on assessments, a reassuring finding, given concern in the literature that values (e.g., around risk and medical expertise) may differ between physicians and patients (42) and findings that physicians' personal values impact on their use of risk-relative standards in capacity assessment (34). We note that capacity guidance (43) and an assessment tool (44) developed in Switzerland include prompts for practitioners to reflect on their personal values and potential bias in relation to the decision at hand.

#### Finding 2—Experienced Assessors Are Aware of Challenges and Pitfalls

The second main finding, related to the last point, is that the liaison psychiatrists we interviewed presented as self-reflective and acutely aware of challenging issues and possible pitfalls in difficult cases. There were several examples of this. Psychiatrists were mindful of the dangers of imposing their own values on their patients, to the extent that one psychiatrist withdrew from capacity assessments on certain subjects (Theme 3). Where decision-making was influenced by extreme emotions or an unusual personality, psychiatrists expressed concern about slipping into paternalism (Theme 1). They frequently considered alternative explanations for unusual decision-making, for example, considering whether an apparent lack of insight might represent a patient minimizing her difficulties due to her strong values (Theme 1), or whether a lack of engagement in assessment might be due to the assessor being perceived as intrusive (Theme 4). Where it was difficult to disentangle illness from cultural or personality factors, psychiatrists were resourceful (and humble) in involving the patient's family and cultural experts to clarify the situation (Theme 1). These examples challenge perceptions in the literature that psychiatry and medicine are paternalistic, unduly biased toward providing treatment (45), privileging patients physical wellbeing above other factors (46), and “assumed to be over-inclusive in its approach to incapacity” (26). They suggest that, far from being knee-jerk paternalists, front line liaison psychiatrists who frequently conduct capacity assessments approach hard cases with nuance and self-reflection.

#### Finding 3—There Are Multiple Strategies to Resolve Difficult Cases

The third main finding is that liaison psychiatrists have developed a myriad of strategies to resolve difficult to judge cases in the general hospital setting, summarized here:

- To resolve difficulties in determining whether the decision is the patient's own or driven by illness, they paid careful attention to potential illness factors and to the patient as an individual, including involving relevant others (Theme 1).
- Where extreme emotions were at play, they advocated delaying the capacity assessment if possible, involving others with different skill sets, or considering pre-emptive action—as in the case of advance directives for needle phobia (Theme 1).
- They worked to clarify the facts of the situation (the relevant information), including the risks, and ensured these were communicated to the patient at interview (Themes 2 and 4).
- Where risks were high, they argued for a more thorough capacity assessment (Theme 2).
- They were vigilant as to the role of their own values in assessment, and where relevant, sought advice from multidisciplinary colleagues, requested second opinion assessments, and in some cases escalated to ethics committees or the courts (Theme 3).
- Where a patient's engagement was limited, they attempted repeat assessments, worked to build rapport, and drew on observations from colleagues (Theme 4).
- In situations of conflict and tension between the patient and treating team, they deployed negotiation and mediation skills. Sometimes this involved advocating for flexible and individualized treatment or giving the patient more control over their care (Theme 4).
- In crisis assessments, they considered psychodynamic explanations for behavior and drew on psychotherapeutic skills to work with ambivalence (Theme 4).
- Finally, they considered whether the real question underlying some requests for capacity assessment was whether it was right to overrule the patient, if incapacitous. They clarified the goals of the assessment and redirected focus where necessary (Theme 4).

### Implications

- Capacity assessments, especially difficult ones, require both clinical and ethical-legal knowledge and skills. There is a danger that assessments using only ethical or legal expertise might miss issues needed to resolve difficulty, for example, subtle illness impairing decision-making, or interpersonal dynamics needing a mediation or psychotherapeutic approach. Similarly, an exclusively clinical focus will not equip an assessor to recognize and navigate difficult ethical quandaries, such as the degree to which the assessment should incorporate values and risks.
- Self-awareness and reflection are an essential part of doing difficult capacity assessments. Assessors must be vigilant about the identified pitfalls, for example, using their own values as a kind of yard stick against which the patient's values might be judged. For difficult cases, further development of diverse, multidisciplinary review mechanisms might be helpful, to ensure the influence of professional values is scrutinized.
- Education and training programmes for practitioners approaching hard capacity assessments should take both clinical and ethical aspects into account. The strategies to resolve difficulty used by psychiatrists in our study could usefully be included in such programme. Further research would be useful to evaluate relevance of such strategies to other practitioners, jurisdictions, and settings outside the general hospital, including social care, mental healthcare, and court proceedings.

### Limitations

- A limitation of this study is that the experiences of liaison psychiatrists interviewed may not be representative of other practitioners who assess capacity. However, our sample was heavily involved in ‘front line' capacity assessment in a direct or supervisory capacity and a substantial proportion also reported ‘extra-curricular' interest in capacity issues. Focusing on this group brings advantages in terms of opportunities for ‘good practice' learning.
- Similarly, cases brought may not be representative of hard cases in other jurisdictions. However, in interviewing psychiatrists applying similar, but not identical, legislation (47–50) (see Supplementary Material 5), we aimed to ensure that challenges encountered were not jurisdiction specific. Of course, assessors must be clear about the legal framework within which they are operating.
- Due to our sample size and choice of qualitative approach, we did not carry out a comparative analysis across jurisdictions. On overall impressions, there were no major differences between types of cases, or resolution approaches, brought across the two larger jurisdictions, however further studies with a comparative focus would be useful.
- The thematic analysis involved interpretation of the transcripts by the researchers, three of whom are psychiatrists, and this shaped how themes were identified and presented. However, the experience of the authors meant they were familiar with the clinical setting and concepts raised by the interviewees.

## Conclusion

In difficult capacity assessments, there is a crucial need for both clinical and ethical knowledge and skills. We hope that our identification of strategies for resolution of hard capacity cases can contribute to training and education of practitioners, and that the usefulness of such strategies can be tested across health and social care settings. In particular, the use of the clinical method to resolve difficult capacity assessments is an important finding which should be explored in further research. Further, our analysis highlights what self-aware and reflective practice can look like. Practitioners assessing capacity should seek to develop this aspect of their practice, and health and social care systems should consider innovative mechanisms to support this. Finally, we hope that our elucidation of what concerns practitioners, with case examples, will provide fruitful material to enlighten normative discussion on thorny conceptual issues such as the role of emotions and risk in decision-making capacity.

## Data Availability Statement

The transcripts underlying this study are not freely available as we have agreed that participants be given the opportunity to review any reports drawing on clinical material to ensure they are satisfied with confidentiality. Further inquiries can be directed to the corresponding author.

## Ethics Statement

The studies involving human participants were reviewed and approved by King's College London Research Ethics Committee (LRS-17/18-4849). The participants provided their written informed consent to participate in this study.

## Author Contributions

NK led on recruitment, interview protocol design, data collection, and analysis, with supervision from AR, GO, and SK. NK wrote the first version of the manuscript. All authors contributed to and approved its final version. All authors contributed to study conceptualization and design.

## Funding

The authors are core researchers in the contested capacity workgroup, part of the Mental Health and Justice Project funded by a grant (203376/2/16/Z) from the Wellcome Trust. For the purpose of open access, the author has applied a CC BY public copyright license to any Author Accepted manuscript version arising from this submission. NK's MD(Res) degree is supported by a grant from Mental Health Research UK and the Schizophrenia Research Fund. She was also recipient of the 2021 Margaret Slack Traveling Fellowship Award from the Royal College of Psychiatrists Faculty of Academic Psychiatry.

## Author Disclaimer

SK's views are his views only and do not represent the views or policies of the NIH, DHHS, or the US Government.

## Conflict of Interest

The authors declare that the research was conducted in the absence of any commercial or financial relationships that could be construed as a potential conflict of interest.

## Publisher's Note

All claims expressed in this article are solely those of the authors and do not necessarily represent those of their affiliated organizations, or those of the publisher, the editors and the reviewers. Any product that may be evaluated in this article, or claim that may be made by its manufacturer, is not guaranteed or endorsed by the publisher.

## Supplementary Material

Supplementary Material 1 Consent form.

Supplementary Material 2 Participant information sheet.

Supplementary Material 3 Topic guide.

Supplementary Material 4 Qualitative reporting checklist.

Supplementary Material 5 Brief summary of relevant capacity legislation.
