# Supplementary material for: Difficult Capacity Cases—The Experience of Liaison Psychiatrists. An Interview Study Across Three Jurisdictions
Source: Front Psychiatry. 2022 Jul 11;13:946234. doi: 10.3389/fpsyt.2022.946234 (PMC9309683; doi:10.3389/fpsyt.2022.946234)
Supplement: Supplementary Material 1 — Consent form. [file Data_Sheet_1.docx]

## Supplementary Material 1. Consent form for Interview Study

**CONSENT FORM FOR PARTICIPANTS IN RESEARCH STUDIES**

**
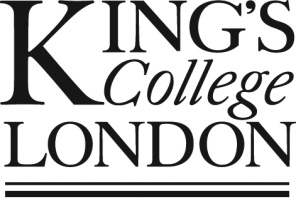
**

**Please complete this form after you have read the Information Sheet and/or listened to an explanation about the research.**

**Title of Study: Contested Capacity Assessment:**

**King’s College Research Ethics Committee Ref:** LRS-17/18-4849

Thank you for considering taking part in this research. The person organising the research must explain the project to you before you agree to take part. If you have any questions arising from the Information Sheet or explanation already given to you, please ask the researcher before you decide whether to join in. You will be given a copy of this Consent Form to keep and refer to at any time.

**Please tick or initial**

I confirm that I understand that by ticking/initialling each box I am consenting to this element of the study. I understand that it will be assumed that unticked/initialled boxes mean that I DO NOT consent to that part of the study. I understand that by not giving consent for any one element I may be deemed ineligible for the study.

**Please tick or initial**

1. I confirm that I have read and understood the information sheet dated 22/06/2018 for the above study. I have had the opportunity to consider the information and asked questions which have been answered satisfactorily.
2. I understand that I will be able to withdraw my data up to 6 months after my interview.
3. I consent to the processing of my personal information for the purposes explained to me. I understand that such information will be handled in accordance with the terms of the General Data Protection Regulation.
4. I understand that my information may be subject to review by responsible individuals from the College for monitoring and audit purposes.
5. Anonymity is optional for this research. Please select from the following 3 options:
   1. I agree to be fully identified
   2. I agree to be partially identified
   3. I wish to remain anonymous
6. I consent to my interview being audio/video recorded.
7. I consent to the use of excerpts from my interview for purposes

of publications and other research outputs

**__________________ __________________ _________________**

**Name of Participant Date Signature**

**__________________ __________________ _________________**

**Name of Researcher Date Signature**
