# Supplementary material for: Difficult Capacity Cases—The Experience of Liaison Psychiatrists. An Interview Study Across Three Jurisdictions
Source: Front Psychiatry. 2022 Jul 11;13:946234. doi: 10.3389/fpsyt.2022.946234 (PMC9309683; doi:10.3389/fpsyt.2022.946234)
Supplement: Supplementary Material 2 — Participant information sheet. [file Data_Sheet_2.docx]

## Supplementary Material 2. Participant information sheet for Interview Study


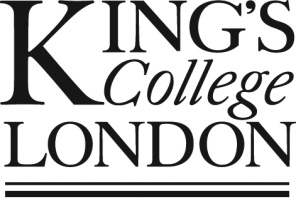
INTERVIEW INFORMATION SHEET FOR PARTICIPANTS

*REC Reference Number:* LRS-17/18-4849

**YOU WILL BE GIVEN A COPY OF THIS INFORMATION SHEET**

**Contested capacity assessment**

We would like to invite you to participate in this original research project. You should only participate if you want to; choosing not to take part will not disadvantage you in any way. Before you decide whether you want to take part, it is important for you to understand why the research is being done and what your participation will involve. Please take time to read the following information carefully and discuss it with others if you wish. Ask us if there is anything that is not clear or if you would like more information.

**What is the purpose of the study?**

This project, which runs from 2018 to 2022, looks at the legal and clinical evidence that makes the assessment of decision-making capacity in a proportion of cases contested or hard. We will draw on that evidence to develop educational interventions and tools to enable more satisfactory resolution of difficult capacity questions.

**Why have I been invited to take part?**

The following people will be invited to participate in this study: (a) psychiatrists with experience in conducting capacity assessments (in particular liaison and old age psychiatrists); (b) lawyers with experience in acting in proceedings concerning mental capacity; (c) current and former judges of the Court of Protection; and (d) (in focus groups) social workers with experience of capacity issues in the community setting.

**Do I have to take part?**

Participation is voluntary. You do not have to take part. You should read this information sheet and if you have any questions you should ask the research team.

**What will happen to me if I take part?**

If you decide to take part you will be given this information sheet to keep and will be asked to sign a consent form. We will then discuss the interview procedure with you and arrange to interview you in a private place (for confidentiality reasons).

The interview will take approximately one hour. It will be recorded, subject to your permission. All recordings of data on audio-equipment will be kept secure (see further below). Even if you have decided to take part, you are still free to stop your participation at any time during the interview and to have research data/information relating to you withdrawn without giving any reason up until 6 months from the date of your interview.

**What form will the interview take?**

The interview will be semi-structured, with two main areas of focus:

1. Cases in which capacity assessment may be hard or contested, including by reference to: (1) the nature of issue in respect of which capacity is contested; (2) the nature of disorder or impairment said to be relevant; and (3) particular aspect(s) of capacity test under the relevant legal framework which cause difficulty. You will be invited to think both generically about these issues and, where relevant, to draw upon your own personal experience.
2. Your experience of different forms of resolution of capacity issues, including outside the court room and within court proceedings

We may share with you preliminary findings from the typology of contested/hard capacity issues that we have drawn up in from analysis of case-law in England, Scotland and New Zealand, both to stimulate discussion and also for you to comment upon.

**What are the possible benefits?**

Your participation includes an opportunity to contribute to the development of tools which will enable the more satisfactory resolution of difficult questions of decision-making capacity.

**Will my taking part be kept confidential?**

Confidentiality and your anonymity, should you wish it, are guaranteed by accordance with the terms of the General Data Protection Regulation.

All information we collect from you, including the recordings, will be kept on a computer. The computer will need a password to open it. Any hard copies of the information will be kept under lock and key at King’s College London. If you have asked for anonymity, when we write down what you have said for purposes of the next stage of the research project, we will take out your name and any identifying details except your professional background.

You may withdraw from this study at any time up until 6 months from the day you participated in this study without consequence. In that case, transcriptions, observations and all information already provided will be destroyed. If you do agree to proceed, you may choose to stop the interview at any time. If you do not wish to answer a particular question or would like to ask us questions, please feel free to stop us so that we can talk about the relevant issue.

**Will patient/service user information be kept confidential?**

It is likely that you will want to discuss real cases (either legal or clinical) that you have encountered that you have found difficult. If that case is not already in the public domain and/or any relevant personal information relating to the patient/service user in question is not in the public domain, we will keep such information confidential, and take steps to ensure that identifying information is removed from any report of the interview that we use for the next stages of the research project. We will show you a draft of any report or any other materials that we propose to use which draws upon information you have provided during the interview so that you can confirm that you are satisfied that relevant information has been removed.

**How is the project being funded?**

The Wellcome Trust (a British funding agency) is funding this research.

**What will happen to the results of the study?**

Study results will be published in academic journals and as book chapters in edited volumes. They will be used in conference and seminar presentations. We will also publish the results in non-academic formats including reports. When research results are published and/or presented, all participants will remain anonymous (unless you want us to use your real name). Final results and publications will also be shared with participants as far as possible.

**Who should I contact for further information?**

If you have any questions or require more information about this study, please contact clinical research associate Nuala Kane using the following contact details. You can also contact us if you have any concerns about the way the study has been conducted, or about your rights as a research participant.

**Dr Nuala Kane**

Department of Psychological Medicine, King’s College London

Institute of Psychiatry, Psychology and Neuroscience

16 De Crespigny Park

Denmark Hill, SE5 8AF

London, UK.

Tel: XXXXXX

Email: nuala.1.kane@kcl.ac.uk

Project website: www.mhj.org.uk

**What if I have further questions, or if something goes wrong?**

If this study has harmed you in any way or if you wish to make a complaint about the conduct of the study you can contact King's College London using the details below for further advice and information:

The Chair, Psychiatry, Nursing & Midwifery Research Ethics Sub-Committee, [pnm@kcl.ac.uk](mailto:pnm@kcl.ac.uk).

**Thank you for reading this information sheet and for considering taking part in this research.**
