# Supplementary material for: Difficult Capacity Cases—The Experience of Liaison Psychiatrists. An Interview Study Across Three Jurisdictions
Source: Front Psychiatry. 2022 Jul 11;13:946234. doi: 10.3389/fpsyt.2022.946234 (PMC9309683; doi:10.3389/fpsyt.2022.946234)
Supplement: Supplementary Material 3 — Topic guide. [file Data_Sheet_3.docx]

## Supplementary Material 3. Full topic guide^1^ for Difficult Capacity Cases study – England and Wales version

| **1. Sample** | 1. Professional background/ work setting |  |
| --- | --- | --- |
|  | 2. Experience of a) capacity assessments and b) difficult or contested cases, in terms of frequency and relevant issues/ decisions |  |
|  | 3. Education/ training on assessing capacity |  |
|  |  |  |
| **2. Introduction** | 4. Why do you think some capacity assessments are more difficult than others? |  |
|  | 5. What sorts of cases (persons, situations) do you find present difficult capacity issues? |  |
|  |  |  |
| **3. Hard Cases**  ***Decision** | 6. Demographics/ presentation of assessed |  |
|  | 7. What was the issue or decision?  What were the available options? |  |
|  | 8. What was the context of the decision?  Was there pressure from time, resources? |  |
|  | 9. How did you view the potential consequences (risk) of the decision? What role, if any, did this play in the assessment? |  |
| ***Assessment** | 10. How did the need for the assessment come about? Time/ resources pressure? |  |
|  | 11. Were there multiple assessments? Was there disagreement between assessors, assessed person or others? |  |
| ***Impairment/ Causative Nexus (other factors may include undue influence or the subject’s personal or cultural worldview)** | 12. Impairment of mind or brain felt to be relevant |  |
|  | 13. How did the impairment affect decision-making? |  |
|  | 14. Was this the only factor affecting the person’s decision-making?  How did other factor(s), if present, interact with the impairment? |  |
| ***Crux of issue** | 15. What made this case hard**?**  If there was disagreement, how do you explain the reason for this? |  |
|  | 16. Was there a particular aspect of the capacity test which caused difficulty? |  |
| ***Wishes and preferences, beliefs and values** | 17. Were you aware of the wishes and preferences of the individual being assessed? Were you aware of their beliefs and values, either personal or cultural? |  |
|  | 18. How did you become aware of these - did the person express these at the time or was there another source? |  |
| ***Practical steps** | 19. Were steps to maximise the person’s capacity taken or considered? On reflection are there practical steps which might have been taken or considered? |  |
| ***Resolution** | 20. How was the case resolved? |  |
|  | 21. Do you know what happened afterward? |  |
|  | 22. Were you satisfied with the resolutions? Were others satisfied e.g. person, family, other stakeholders? How did they proceed if unsatisfied? |  |
|  | 23. Specific questions as relevant:  Was the assessment deferred or carried out over multiple sessions? Was knowledge of person’s ‘real world’ decision-making taken into account, and how? Was information sought from other sources to aid the assessment e.g. from family members, previous wishes, advance statements? Were colleagues/ others present or consulted? |  |
|  | | |
| **4. Objectivity/ Defensibility** | 24. How objective do you think capacity assessments are? What does objectivity mean to you? Is it something desirable? |  |
|  | 25. What do you think constitutes a satisfactory or defensible resolution to a difficult capacity assessment? |  |
|  | 26. Has a capacity assessment ever caused you to reflect on your own values? |  |
|  | 27. What processes or safeguards could or should compensate for human fallibility or subjectivity in assessment? |  |
|  |  |  |
| **5. Presumption of Capacity** | 28. What role does the presumption of capacity play in capacity assessments you are involved in? |  |
|  | | |
| **6. Functional abilities^2^** | 29. In your own words, what do you think it means… to understand? |  |
|  | 30. To retain? |  |
|  | 31. To use or weigh? |  |
|  | 32. To communicate? |  |
|  | 33. How does one fail these abilities? Does this vary depending on the disorder or the decision at hand? |  |
|  | 34. Are there other factors you find relevant to capacity assessment aside from the named MCA abilities? |  |
|  |  |  |
| **7. Unwise decisions** | 35. What is the difference between an unwise decision and inability to use/ weigh? |  |
|  | 36. For unwise decisions what assessment findings would allow a person to ‘pass’ the assessment? |  |
|  |  |  |
| **8. The support principle** | 37. Can you think of examples where practical steps to maximise capacity made a big difference? |  |
|  |  |  |
| **9. Court Resolutions** | 38. Have you had any experience of cases brought to court? Or cases you felt should have gone to court but did not? |  |
|  |  |  |
| **10. Improvement/ Education** | 39. What could be done to increase satisfactory resolutions of hard or contested capacity cases? |  |
|  | 40. What education or training would help assessors in approaching difficult cases? |  |

^1^Our interview protocol was broad and not all components of the interviews are analysed in this study.

^2^Note that this protocol was adapted for psychiatrists from Scotland and New Zealand to reflect their capacity legislation.
