# Supplementary material for: Difficult Capacity Cases—The Experience of Liaison Psychiatrists. An Interview Study Across Three Jurisdictions
Source: Front Psychiatry. 2022 Jul 11;13:946234. doi: 10.3389/fpsyt.2022.946234 (PMC9309683; doi:10.3389/fpsyt.2022.946234)
Supplement: Supplementary Material 5 — Brief summary of relevant capacity legislation. [file Data_Sheet_5.docx]

## Supplementary Material 5. Brief summary of capacity legislation relevant to Difficult Capacity Cases study

Mental Capacity Act (2005), England and Wales

*A person lacks capacity in relation to a matter if at the material time he is unable to make a decision for himself in relation to the matter because of an impairment of, or a disturbance in the functioning of, the mind or brain […] A person is unable to make a decision for himself if he is unable (a) to understand the information relevant to the decision, (b) to retain that information, (c) to use or weigh that information as part of the process of making the decision, or (d) to communicate his decision.*

Adults with Incapacity (Scotland) Act (2000), Scotland

*“Incapable” means incapable of (a) acting; or (b) making decisions; or (c) communicating decisions; or (d) understanding decisions; or (e) retaining the memory of decisions, as mentioned in any provision of this Act, by reason of mental disorder or of inability to communicate because of physical disability.*

Protection of Personal and Property Rights Act (1988), New Zealand

The PPPR Act has multiple capacity tests, helpfully summarised by The Law Foundation, New Zealand:

“The Act provides no single test for incapacity, which makes it complex legislation to follow and apply. In general terms, however, the Act says a person lacks capacity if they do not understand the nature or cannot foresee the consequences of decisions, or are unable to communicate them.”(50)
